# The effect of desloratadine on ischemia reperfusion induced oxidative and inflammatory renal injury in rats

**DOI:** 10.1080/0886022X.2020.1769656

**Published:** 2020-06-11

**Authors:** Huseyin Kocaturk, Fevzi Bedir, Mehmet Sefa Altay, Ebubekir Bakan, Bahadir Suleyman, Gulce Naz Yazici, Mukadder Sunar, Zeynep Suleyman, Halis Suleyman

**Affiliations:** a Department of Urology, Health Sciences University, Erzurum Regional Training and Research Hospital, Erzurum, Turkey; b Department of Biochemistry, Faculty of Medicine, Ataturk University, Erzurum, Turkey; c Department of Pharmacology, Faculty of Medicine, Erzincan Binali Yildirim University, Erzincan, Turkey; d Department of Histology and Embryology, Faculty of Medicine, Erzincan Binali Yildirim University, Erzincan, Turkey; e Department of Anatomy, Faculty of Medicine, Erzincan Binali Yildirim University, Erzincan, Turkey; f Department of Nursing, Faculty of Health Sciences, Erzincan Binali Yildirim University, Erzincan, Turkey

**Keywords:** Desloratadine, ischemia, reperfusion, renal, rat

## Abstract

**Purpose:**

To examine the effect of desloratadine on kidney ischemia-reperfusion (I/R) injury in albino Wistar male rats using biochemical and histopathological methods.

**Methods:**

The treated with ischemia–reperfusion + 5 mg/kg desloratadine (IRD) group (*n*–6) was given 5 mg/kg desloratadine by gavage orally, and applied renal ischemia-reperfusion (BIR) group (*n*–6) and control (SG) group undergoing Sham operation (*n*–6) rats were given distilled water as solvent one hour before ketamine anesthesia. During the anesthesia period, ischemia was induced for 2 h unilaterally in the left kidney of all rats followed by reperfusion for 6 h. The kidneys of the SG group had sham operation without any intervention.

**Results:**

Our biochemical test results showed that malondialdehyde (MDA), nuclear factor kappa (NF-κB), tumor necrosis factor alpha (TNF-α), interleukin one beta (IL-1β), creatinine, and blood urea nitrogen (BUN) levels were significantly increased in the BIR group compared to the healthy control and IRD groups treated with desloratadine. Histopathological results were revealed tubular dilatation, tubular necrosis, loss of brushy margins, cast formation, and apoptotic bodies in tubular epithelial cells in the BIR group. There were no histopathological findings except for the swelling of tubule epithelial cells and the accumulation of proteinous material in some tubule lumens in renal tissue of desloratadine-treated rats.

**Conclusions:**

Experimental results suggested that desloratadine may be useful in the treatment of renal I/R injury.

## Introduction

Ischemia is a condition where the tissue is deprived of oxygen when there is a decrease or completely no blood flow to the tissue [1]. In tissue ischemia, a series of chemical events progressing to cellular dysfunction and cell necrosis are seen. Therefore, the first intervention in ischemic tissue is to ensure reperfusion of the tissue [2]. Reoxygenation in reperfusion leads to the formation of reactive oxygen species (ROS) [3]. These ROS are known as reperfusion mediators oxidize cell membrane lipids and they, produce toxic products such as aldehyde and malondialdehyde (MDA) from lipids [4]. ROS also increases proinflammatory cytokine nuclear factor-κB (NF-κB) production [5]. NF-κBs have been shown to stimulate the secretion of proinflammatory interleukin 1 beta (IL-1β), tumor necrosis factor alpha (TNF-α), and other inflammation mediators [6]. Briefly, ischemia-reperfusion (I/R) injury is a complex pathological process that begins with deoxygenation of tissue and continues with the production of free oxygen radicals and expands with the inflammatory response [2]. The relevant literature data showed the value of antioxidant and anti-inflammatory therapy in I/R tissue damage.

Desloratadine to be examined in this study for its protective effect against kidney I/R injury, is a potent and non-sedative H_1_ histamine receptor antagonist used in the symptomatic treatment of urticaria and allergic rhinitis [7]. Desloratadine has been reported to antagonize the increase of MDA, the final product of lipid peroxidation, and to reduce endogenous antioxidant glutathione (GSH) in the allergy model [8]. Roumestan *et al.* showed that there was a desloratadine inhibited NF-κB production whereas [9] Wu *et al.* argued that desloratadine inhibited both basal and histamine-induced NF-κB [10]. This data suggested that desloratadine can protect kidney tissue from I/R damage. There is no study in the literature examining the protective effect of desloratadine against renal I/R injury. Therefore, the aim of this study was to examine the effect of desloratadine on kidney I/R injury in albino Wistar male rats using biochemical and histopathological methods.

## Materials and methods

### Animals

Eighteen male albino Wistar rats weighing between 280-295 g were used in this study. The animals were obtained from Ataturk University Medical Experimental Application and Research Center. Animals were kept and fed at room temperature (22 °C) in the laboratory where the study to be performed in order to let the animals, adapt to the environment.

### Chemicals

Ketamine was obtained from Pfizer (Turkey) and desloratadine was obtained from Sanofi Pharmaceuticals Industry and Commerce (Turkey).

### Experimental Groups

The animals were divided into three groups: BIR group applied renal I/R, IRD group treated with I/*R* + 5 mg/kg desloratadine, and control group (SG) undergone Sham operation.

### Anesthesia

Surgical procedures on rats were performed under sterile conditions 60 mg/kg ketamine was administered as the anesthesia intraperitoneally (i.p.) and 0.6 mg/kg xylazine (inhaler) was given at appropriate intervals. After the ketamine injection, the rats were allowed to wait for the appropriate period for surgical intervention. The period in which the animals were immobilized in the supine position was considered as the appropriate anesthesia period for surgical intervention [11].

### Surgical and pharmacological procedures

One hour before ketamine anesthesia, the IRD group (*n*–6) was orally given 5 mg/kg desloratadine by gavage, and BIR (*n*–6) and SG (*n*–6) rats were given distilled water as solvent. During the anesthesia period, left kidneys of all rats were opened unilaterally through the dorsal section. After that, ischemia was induced for two hours by placing clips on the renal artery and veins coming to the left kidney of IRD and BIR group rats. No procedure was performed on the left kidneys of the SG group rats and the incision was sutured. Then, reperfusion was performed in IRD and BIR groups for 6 h. IRD and BIR groups were sacrificed with a high dose of anesthesia and their kidneys were removed. Blood was drawn intracardiacly before animals were sacrificed with high-dose anesthesia for BUN and creatinine measurements. Biochemical and histopathological examinations were performed on the extracted kidneys. Biochemical and histopathological results obtained from the IRD and SG groups were compared with those obtained from the BIR group’s left kidneys.

### Biochemical analyses

#### MDA Assessment

The method used by Ohkawa *et al.* was taken as basis for MDA assessment [12]. This method is based on the spectrophotometric assessment of the absorption of the pink complex created by thiobarbituric acid (TBA) and MDA at high temperature (95 °C) at 532 nm wavelength. Homogenates were centrifuged for 20 min at 5000 g and these supernatants were used in the determination of the amount of MDA. 250 μl homogenates, 100 μl 8% sodium dodecyl sulfate (SDS), 750 μl 20% acetic acid, 750 μl 0.08% TBA and 150 μl distilled water were vortexed into capped test tubes through pipette. The mixture was incubated for 60 min at 100 °C. 2.5 mL *n*-butanol was added to the mixture and then, spectrophotometric analysis was performed. The amounts of resultant red color were measured using 3 mL cuvettes at 532 nm. MDA amounts of the samples were determined by using the standard graphics formed with the MDA stock solution which was previously prepared considering the dilution coefficients.

#### tGSH assessment

The DTNB [5,5′-Dithiobis (2-nitrobenzoic acid)] in the assessment environment is a disulphide chromogen and is easily reduced by sulfhydryl group compounds. The resultant yellow color was spectrophotometrically assessed at 412 nm[13]. Homogenates were centrifuged for 10 min at 12,000 g and the supernatants were used in the determination of the amount of MDA. 250 μl measuring buffer (200 mM Tris–HCl, pH = 8.2 involving 0.2 mM EDTA), 500 μl supernatant, 100 μl 5,5′-Dithio-bis (2-nitrobenzoic acid) (DTNB) and 7900 μl methanol were vortexed into capped test tubes through pipette. The mixture was incubated for 30 min at 37 °C and then, spectrophotometric analysis was conducted. The amounts of resultant yellow color were measured using 3 mL quartz cuvettes at 412 nm. GSH amounts of the samples were determined by using the standard graphics formed with the GSH stock solution which was previously prepared considering the dilution coefficients.

### NF-κB, TNF-α, and IL-1β analysis

Tissue-homogenat NF-κB and TNF-α concentrations were measured using rat-specific sandwich enzyme-linked immunosorbent assay. Rat NF-κB ELISA immunoassay kits (Cat. No:201-11-0288, SunRed). Rat TNF-α and Rat IL-1β ELISA kits (Cat no: YHB1098Ra, Shanghai LZ). Analyses were performed according to the manufacturers’ instructions. Briefly, monoclonal antibody specific for rat NF-κB, TNF-α, and IL-1β were coated onto the wells of the micro plates. The tissue homogenat, standards, and biotinylated monoclonal antibody specific and streptavidin-HRP (Horseradish peroxidase) were pipetted into these wells and then incubated at 37 °C for 60 min. After washing, chromogen reagent A and chromogen reagent B were added, which is acted upon by the bound enzyme to produce a color. It was incubated at 37 °C for 10 min. Than stop solution was added. The intensity of this colored product is directly proportional to the concentration of rat NF-κB, TNF-α, and IL-1β present in the original specimen. At the end of the course, the well plates were read at 450 nm.The absorbance of the samples was calculated with formulas that used standard graphics.

### Creatinine measurement

Quantitative determination of serum creatinine was performed by spectrophotometric method in Roche cobas 8000 autoanalyzer. This kinetic colorimetric test was based on the Jaffe method. In alkaline solution, creatinine formed a yellow-orange complex with picrate. This complex was measured at a wavelength of 505 nm. The rate of dye formation was proportional to the creatinine concentration in the sample. In the test, ‘rate-blanking’ was used to minimize bilirubin interference. Serum or plasma results were corrected with 26 μmol/L (-0,3 mg/dL) in order to correct the nonspecific reaction of serum/plasma pseudo-creatinine chromogens, including proteins and ketones. Creatinine + picric acid → (Alkaline pH) Yellow-orange complex.

### BUN measurement

Quantitative determination of serum urea level was performed by spectrophotometric method in Roche cobas 8000 autoanalyzer. The following formula was used: BUN = UREA * 0.48. In kinetic testing with urease and glutamate dehydrogenase, urea is hydrolyzed by urease and ammonium and carbonate were formed. Urea + 2H_2_O → (Urease) NH_4_^+^ + CO_3_^2−^. In the second reaction 2-oxoglutarate reacted with ammonium to form L-glutamate when there was glutamate, dehydrogenase (GLDH) and coenzyme NADH (Nicotinamide adenine dinucleotide reduced version) in the medium. For each mole of urea hydrolyzed in this reaction, two moles of NADH are oxidized to NAD^+^. NH_4_^+^ + 2-oxoglutarat + NADH → ^(GLDH)^ L-glutamate + NAD^+^ + H_2_O.The rate of decrease in NADH concentration was directly proportional to the urea concentration in the sample and was measured at 340 nm wavelength.

### Histopathological examination

Renal tissues were fixed in 10% neutral buffered formalin solution. After routine tissue follow up, 5 µm thick sections were stained with hematoxylin–eosin (H&E) and Periodic Acid - Schiff (PAS) dye. Histopathological examination was performed on a light microscope (Olympus BX 51, Tokyo, Japan) by a pathologist not informed about the treatment given to the animals. The photographs were taken with a digital camera (ZEISS Axiocam ICc 5). The entire cortex and medulla, especially the external medulla being the most susceptible to ischemia in the kidney were examined in terms of ischemic damage. Renal sections were examined for tubular necrosis, intratubular cast formation, the presence of apoptotic body, tubular dilatation, and brushy margin loss. The severity of histopathological findings was scored between 0 and 3 (0-normal, 1-mild damage, 2-moderate damage, and 3-severe damage) in each section.

### Statistical analyses

Results of the experiment were expressed as ‘mean value ± standard deviation’ (x ± SD). The degree of significance of intergroup differences was determined by one-way ANOVA test. Tukey post hoc test were carried out in the follow up process. While comparing histopathological grades between groups Kruskal–Wallis test was used. Dunn’s test was used for post hoc analysis with bonferroni corrected *p* values. All statistical operations were performed on ‘IBM SPSS for Windows, 22.0’ statistics program and *p* < 0.05 was considered to be significant.

## Results

### MDA and tGSH analysis results

**Figure 1.**
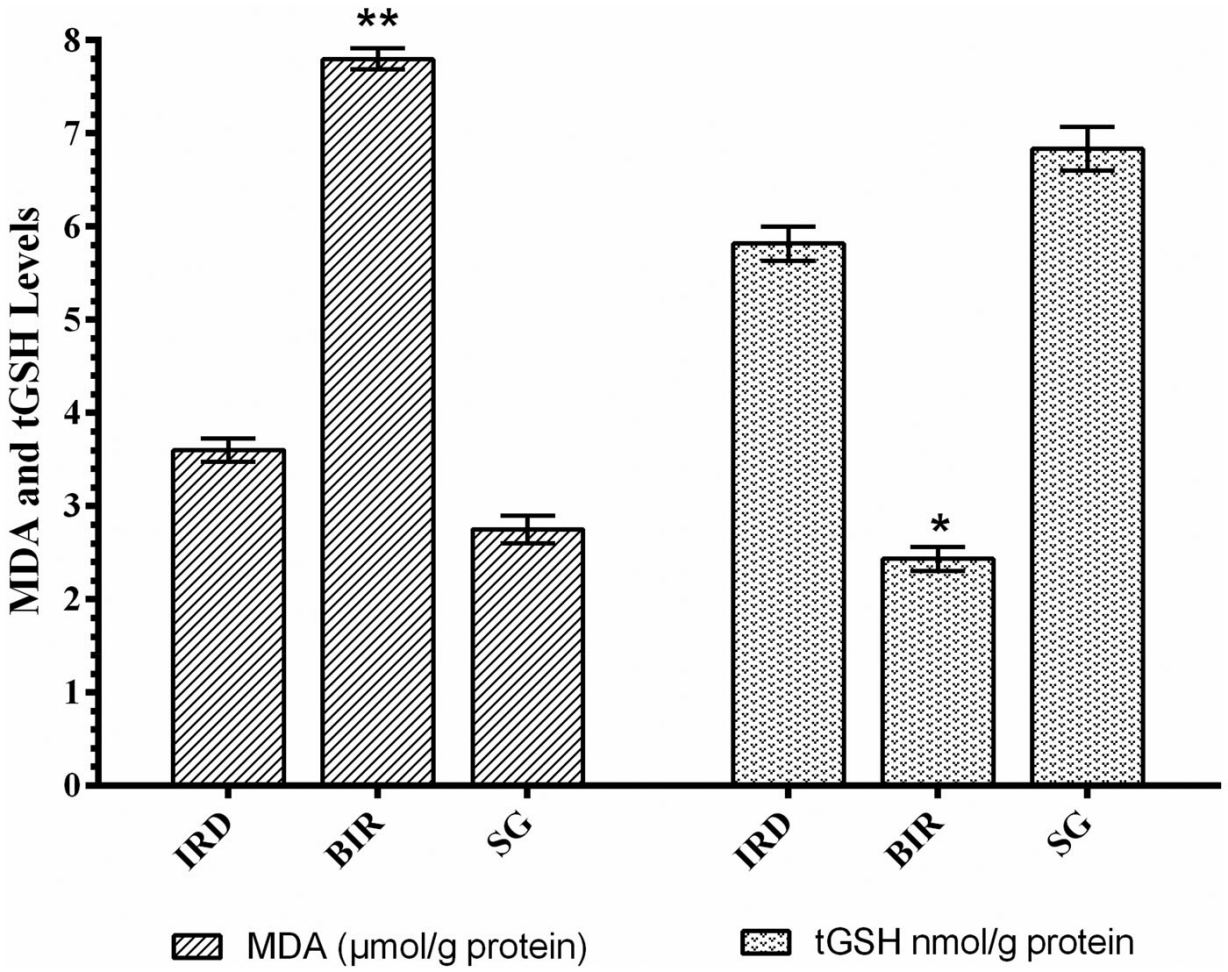
MDA and tGSH levels in the renal tissue of study groups. **p < 0.0001, *p < 0.001 according to SG and IRD groups (*n* = 6).

MDA production increased significantly in kidney tissue of animals treated with I/R compared to Sham group and desloratadine-treated IRD group (*p* < 0.0001). MDA levels in the desloratadine group were similar to that of the Sham group. The difference in MDA levels between desloratadine and Sham groups was statistically insignificant (*p* > 0.05). In addition, the I/R procedure caused a decrease in tGSH in the kidney tissue. tGSH levels in the Sham and Desloratadine groups were significantly higher compared to the I/R group (*p* < 0.001) (Figure 1).

### NF-κB, TNF-α, and IL-1β analysis results

**Figure 2.**
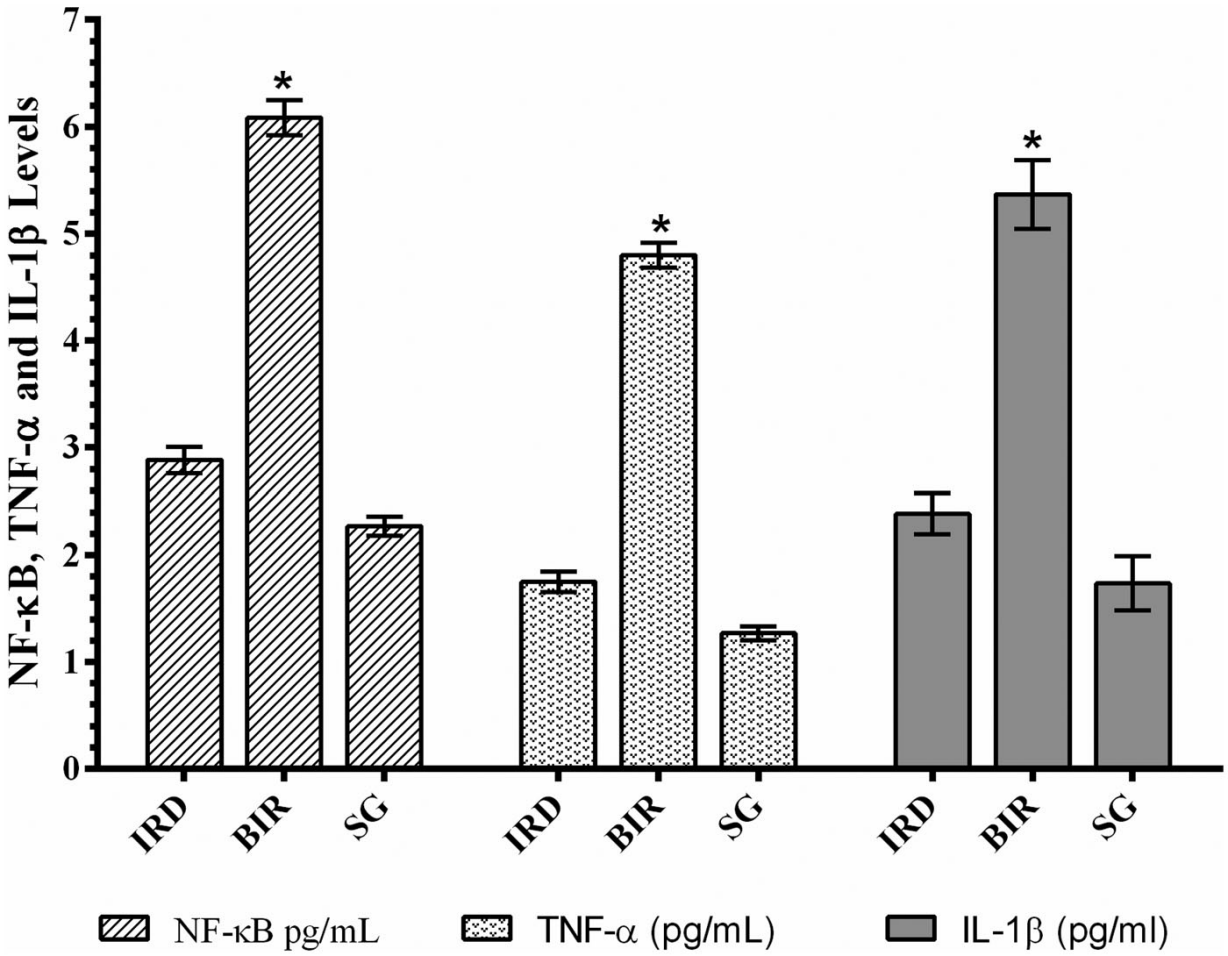
NF-κB, TNF-α, and IL-1β levels in the testis tissue of study groups. *p < 0.0001 according to SG and IRD groups (*n* = 6).

As can be seen in Figure 2, I/R procedure significantly increased NF-κB in the kidney tissue of animals compared to the Sham and desloratadine group (*p* < 0.0001). The difference in NF-κB level between the Sham and desloratadine groups was found to be insignificant (*p* > 0.05). In addition, I/R procedure significantly increased TNF-α and IL-1β production in renal tissue compared to the Sham and desloratadine groups (*p* < 0.0001). The difference in TNF-α and IL-1β levels between the Sham and desloratadine groups was found to be insignificant (*p* > 0.05).

### Creatinine and BUN analysis results

**Figure 3.**
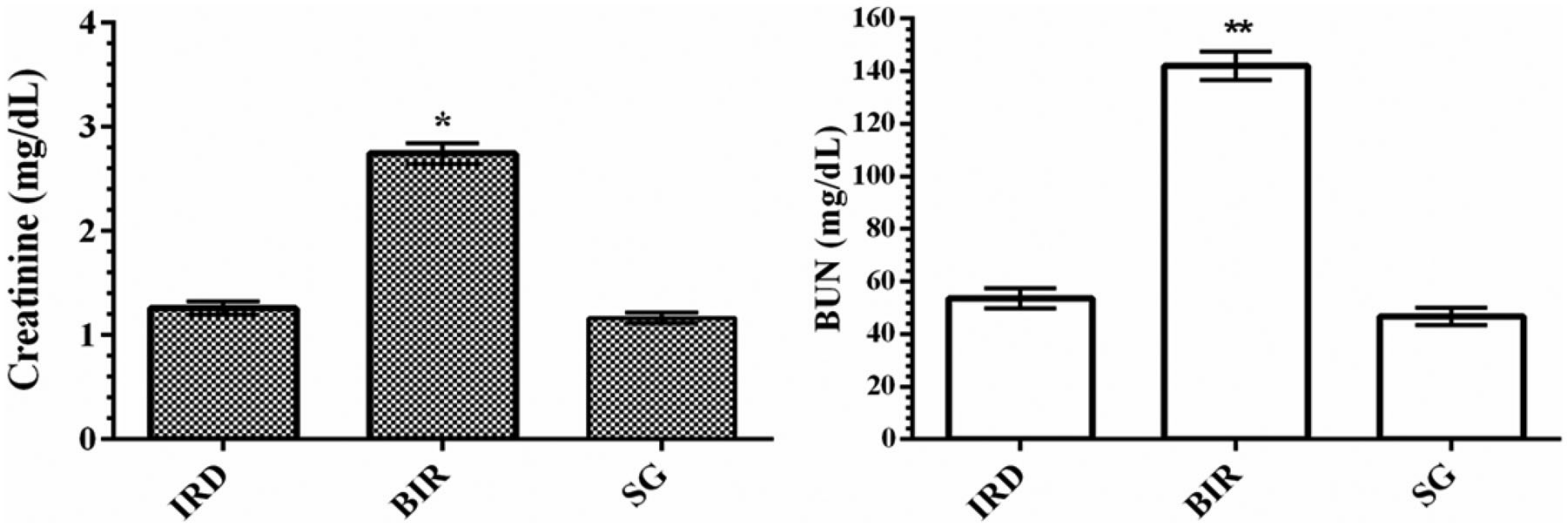
Creatinine and BUN levels in the blood serum of study groups. *p < 0.001 according to SG and IRD groups (*n* = 6). **p < 0.0001 according to IR group (*n* = 6).

As can be seen in Figure 3, I/R procedure performed on the left kidney of the animals increased blood serum creatinine levels compared to the Sham and desloratadine groups (*p* < 0.001). Creatinine levels were found to be similar in the Sham and desloratadine groups, and the difference was statistically insignificant (*p* > 0.05). I/R-associated kidney injury increased BUN levels in the blood serum of animals compared to the Sham and desloratadine groups (*p* < 0.0001). The difference between Sham and desloratadine groups was statistically insignificant (*p* > 0.05).

### Histopathological findings

**Figure 4.**
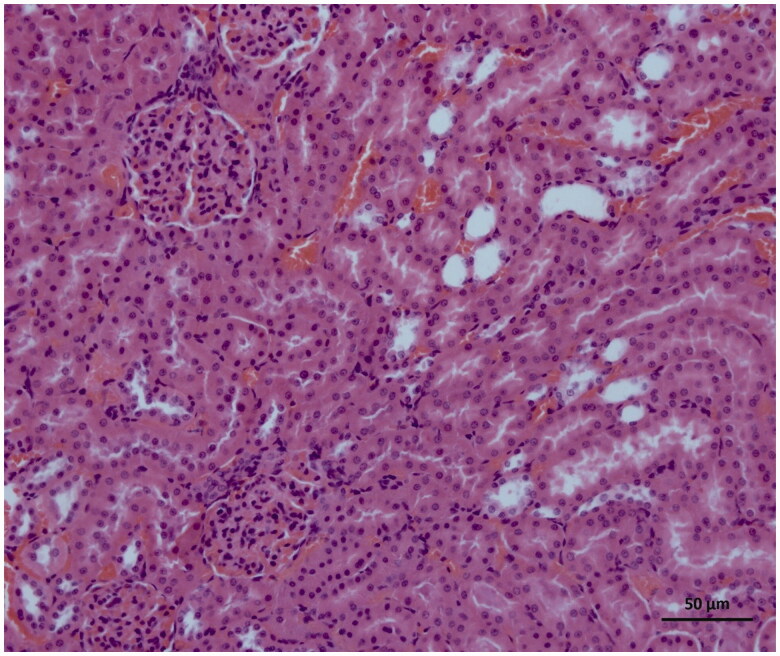
Histology of normal kidney tissue in the Sham group.

**Figure 5.**
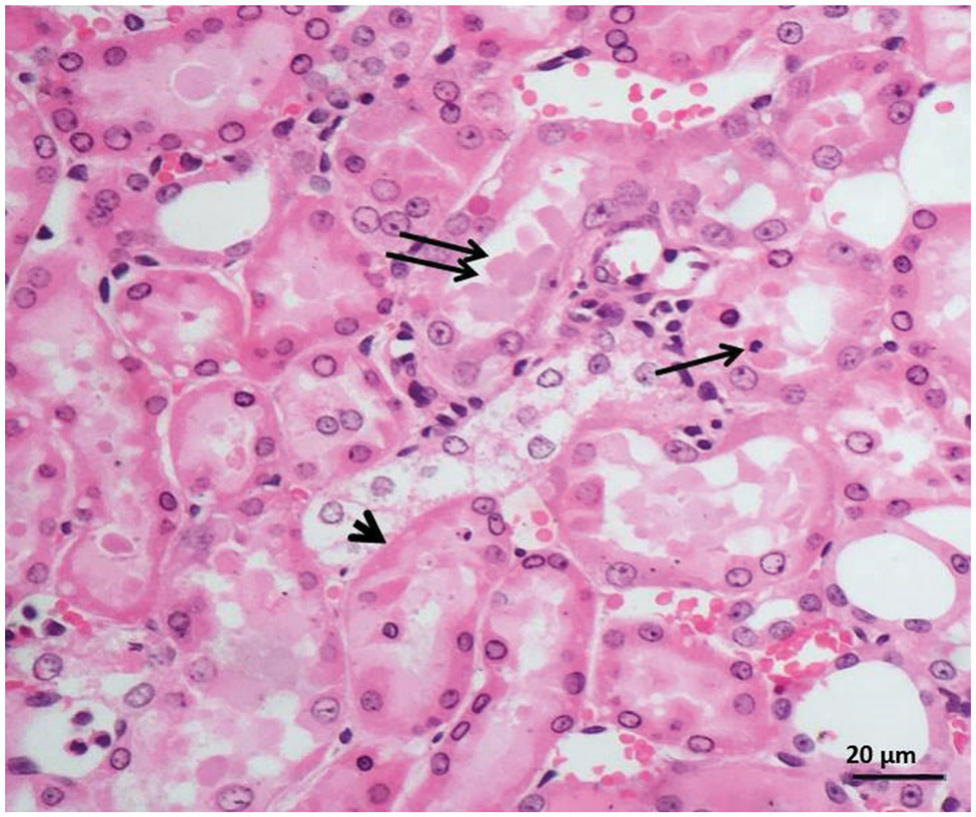
Histopathological results showing tubular dilatation, tubular necrosis (arrowhead), intratubular cast formation (double arrow), apoptotic bodies in tubular epithelial cells (single arrow) in kidney tissue of I/R-treated animals.

**Figure 6.**
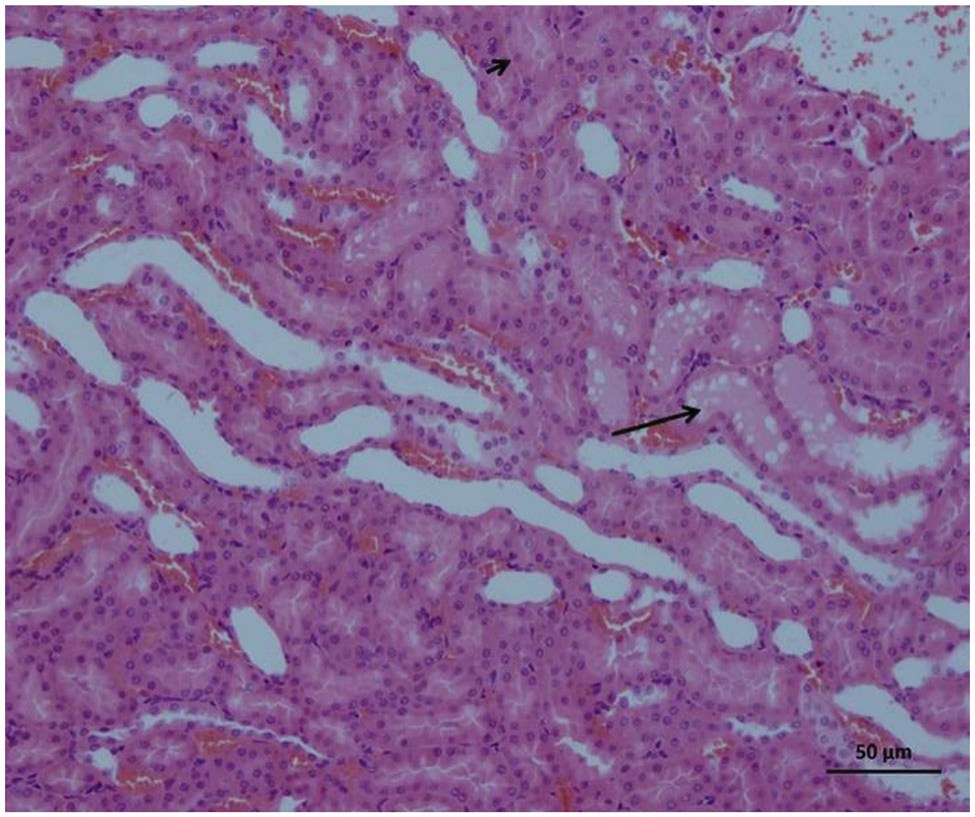
Histopathological results showing swelling in tubular epithelial cells (short arrow) and accumulation of proteinous material (arrow) in some tubule lumens in rats treated with I/*R* + 15 mg/kg desloratadine.

**Figure 7.**
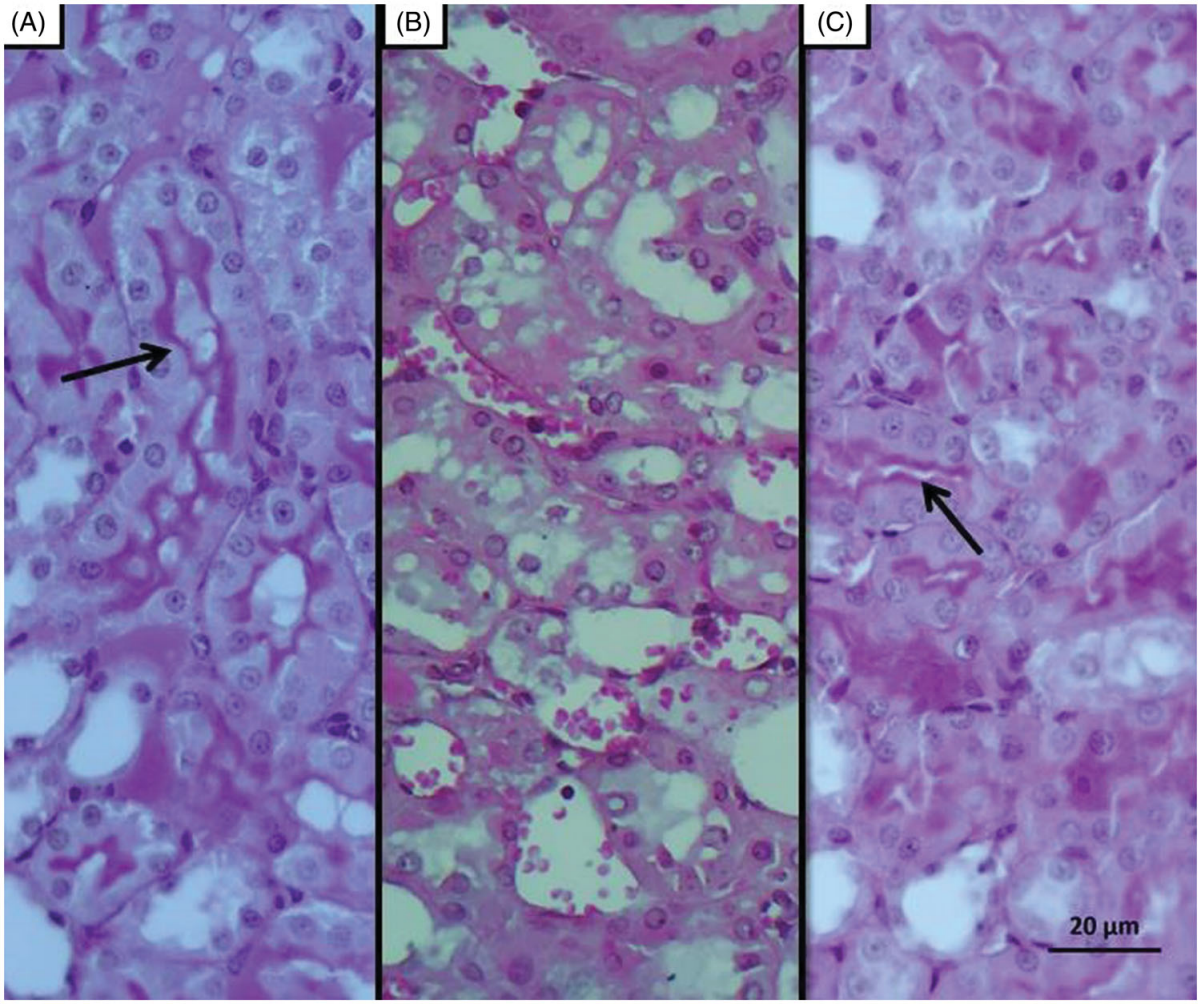
PAS staining results; Histopathology showing loss of brushy margins in proximal tubules in the I/R group (B). Regular appearance of brushy margins (arrow) in the Sham (A) and desloratadine-treated groups (C).

As can be seen in Figure 4, microscopic evaluation revealed no histopathological findings in kidney tissue of Sham group rats. However, significant dilatation, tubular necrosis, intratubular cast formation and apoptotic bodies in tubular epithelial cells were observed in the kidney tissue of rats treated with I/R procedure (Figure 5). There were no histopathologic findings (tubular necrosis, apoptotic bodies in the tubule epithelial cells) except for swelling of tubule epithelial cells and accumulation of proteinous material in some tubule lumens in kidney tissue of desloratadine-treated rats (Figure 6). The results of histopathological examination with PAS dye showed loss of brushy margins in proximal tubules in the I/R group (B). However, no loss of brushy margins was observed in the proximal tubules in the Sham (A) and desloratadine-treated group (C) (Figure 7). The severity of histopathological findings was scored in Table 1.

**Table 1.** The degree of histopathological findings.

	IRD	BIR	SG	p*	*p* ^a^	*p* ^b^	*p* ^c^
TN	0(0-0)	3(3-3)	0(0-0)	<0.001	0.001	1.000	0.001
ICF	1(0-1)	3(2-3)	0(0-0)	0.001	0.047	0.501	<0.001
AC	0(0-0)	3(3-3)	0(0-0)	<0.001	0.001	1.000	0.001
TD	0(0-1)	3(3-3)	0(0-0)	<0.001	0.004	1.000	0.001
PTFK	0(0-0)	3(3-3)	0(0-0)	<0.001	0.001	1.000	0.001

Results were presented as median(minimum-maximum).

AC: apoptotic bodies; ICF: intratubular cast formation; PTFK: proximal tubule brushy edge loss; TD: tubular dilatation; TN: tubular necrosis.

*Kruskal Wallis test was used. Dunn’s test was used for pairwise comparisons of ^a^IRD-BIR, ^b^IRD-SG, ^c^BIR-SG.

0 – normal, 1 – mild damage, 2 – moderate damage and 3 – severe damage.

## Discussion

The protective effect of desloratadine against renal I/R injury in rats was examined biochemically and histopathologically in the study. I/R injury is the main cause of acute kidney injury with increased mortality and chronic kidney disease risks in clinical practice. In the literature, it is clearly summarized that renal I/R injury is a pathological event beginning with ischemia, continues with oxidative stress, and expands with inflammation [11]. Thus, the treatment for the etiopathogenesis of I/R injury is not limited to providing tissue reperfusion. The data suggests that antioxidant and anti-inflammatory therapy during ischemia and post-ischemia reperfusion period is an important treatment option against the pathogenesis of I/R. Our biochemical test results showed that MDA levels in the kidney were higher and tGSH levels were lower in the I/R group compared to the Sham and desloratadine groups. These results indicate that the I/R process alters the oxidant/antioxidant balance in a kidney tissue in favor of oxidants. Furthermore, it has been suggested that ROS production increased in renal tissue treated with I/R. Literature data shows that ROSs, known as reperfusion mediators, oxidize cell membrane lipids and produce toxic products such as MDA from lipids [4]. The reason for the decrease in the amount of tGSH in the I/R group is that the antioxidants become insufficient to neutralize oxidants and that they are over-spent [14]. This is reported to be caused by GSH chemically detoxifying hydrogen peroxide or organic oxides [15]. Recent studies also show that tGSH decreases parallel to MDA increase in renal I/R injury [16].

In this study, it was also found that there was a significant increase in NF-κB, TNF-α, and IL-1β levels in I/R-treated kidney tissue with high MDA levels and low tGSH levels. Previous studies have also stated that ROS increased NF-κB production [5]. In addition, it has been emphasized that NF-κB stimulates the secretion of IL-1β, TNF-α, and other inflammation mediators [6]. NF-κB is known as a transcription factor for the expression of a range of proinflammatory cytokines playing a critical role in the regulation of inflammatory and immune responses [17]. In the study of Aksu U *et al.*, which supports our experimental results, it has been reported that the I/R event causes an increase in oxidative stress and inflammation parameters in kidney tissue and decreases the levels of antioxidants [18]. The high levels of creatinine and BUN in the blood serum of BIR group, where oxidative stress and inflammatory markers increased, compared to the Sham and desloratadine groups were also consistent with the results of recent studies [16,18]. In the experimental studies of Yapanoglu et al., serum creatinine and BUN were used to evaluate oxidative kidney damage and the increase in these parameters (creatinine, BUN) was associated with oxidative damage [19]. In another study, it was reported that increased creatinine and BUN were associated with increased oxidative stress and inflammatory cytokines [20]. The majority of the cases with acute renal failure are due to acute tubular necrosis syndrome. The syndrome usually begins with an acute injury of proximal renal tubular epithelial cells caused by ischemic or nephrotoxic events. This is followed by progressive and often rapid increases in serum BUN and creatinine concentration [21].

There are no studies examining the protective effect of desloratadine against I/R damage in the literature. In the present study, desloratadine which was examined for its possible protective effect against renal I/R injury was found to inhibit the increase of I/R associated increase in MDA, NF-κB, TNF-α and IL-1β production and tGSH depletion in renal tissue. Desloratadine also significantly inhibited the increase of creatinine and BUN in the blood serum of I/R-treated animals. These results indicate that desloratadine suppresses I/R related oxidative stress and inflammation. Sakat MS *et al.* showed that desloratadine inhibited the increase in MDA and proinflammatory cytokine production [22]. Desloratadine has been reported to inhibit MDA overproduction and GSH consumption in damaged tissue[8]. In addition to oxidants, proinflammatory cytokines such as NF-κB, TNF-α, and IL-1β are known to play an important role in the pathogenesis of I/R injury [23,24]. Desloratadine inhibits both basal and histamine-stimulated NF-κB [10], indicating that the production of other proinflammatory cytokines is also inhibited. In a study supporting this idea, it has been reported that NF-κB stimulates the secretion of IL-1β, TNF-α, and other inflammation mediators [6].

The results of biochemical experiments in the present study were consistent with the histopathological findings. Increased tubular dilatation, tubular necrosis, intratubular cast formation, and apoptotic bodies in tubular epithelial cells were observed in renal tissue of the BIR group where oxidant and proinflammatory markers were elevated. Significantly inhibiting the increase in oxidant and proinflammatory markers and the decrease in antioxidants, desloratadine treatment prevented tubular necrosis, formation of apoptotic bodies in tubular epithelial cells, and loss of brushy edges in tubules and mitigated histopathological damage. Jiang et al. [25] showed that tubular dilatation developed in experimental renal I/R injury model. Topdağı et al. [26] reported that necrosis was also developed in renal I/R injury in addition to tubular dilatation. In their study, Feng et al. stated that I/R injury caused degeneration of tubular structures including intratubular cast formation and loss of brushy margins [27]. These literature data support that I/R procedure causes damage to kidney tissue and desloratadine mitigates the damage. In conclusion, I/R procedure performed on the renal artery caused serious damage to kidney tissue. Desloratadine significantly reduced I/R-associated kidney damage. Our experimental results suggest that desloratadine may be useful in the treatment of renal I/R injury.
